# Attenuated Inflammatory Response in Triggering Receptor Expressed on Myeloid Cells 2 (TREM2) Knock-Out Mice following Stroke

**DOI:** 10.1371/journal.pone.0052982

**Published:** 2013-01-03

**Authors:** Matthias W. Sieber, Nadine Jaenisch, Martin Brehm, Madlen Guenther, Bettina Linnartz-Gerlach, Harald Neumann, Otto W. Witte, Christiane Frahm

**Affiliations:** 1 Hans Berger Department of Neurology, Jena University Hospital, Jena, Germany; 2 Neural Regeneration Group, Institute of Reconstructive Neurobiology, University Hospital Bonn, Bonn, Germany; Kaohsiung Chang Gung Memorial Hospital, Taiwan

## Abstract

**Background:**

Triggering receptor expressed on myeloid cells-2 (TREM2) is a microglial surface receptor involved in phagocytosis. Clearance of apoptotic debris after stroke represents an important mechanism to re-attain tissue homeostasis and thereby ensure functional recovery. The role of TREM2 following stroke is currently unclear.

**Methods and Results:**

As an experimental stroke model, the middle cerebral artery of mice was occluded for 30 minutes with a range of reperfusion times (duration of reperfusion: 6 h/12 h/24 h/2 d/7 d/28 d). Quantitative PCR (qPCR) revealed a greatly increased transcription of TREM2 after stroke. We subsequently analyzed the expression of pro-inflammatory cytokines, chemokines and their receptors in TREM2-knockout (TREM2-KO) mice via qPCR. Microglial activation (CD68, Iba1) and CD3-positive T-cell invasion were analyzed via qPCR and immunohistochemistry. Functional consequences of TREM2 knockout were assessed by infarct volumetry. The acute inflammatory response (12 h reperfusion) was very similar between TREM2-KO mice and their littermate controls. However, in the sub-acute phase (7 d reperfusion) following stroke, TREM2-KO mice showed a decreased transcription of pro-inflammatory cytokines TNFα, IL-1α and IL-1β, associated with a reduced microglial activity (CD68, Iba1). Furthermore, TREM2-KO mice showed a reduced transcription of chemokines CCL2 (MCP1), CCL3 (MIP1α) and the chemokine receptor CX3CR1, followed by a diminished invasion of CD3-positive T-cells. No effect on the lesion size was observed.

**Conclusions:**

Although we initially expected an exaggerated pro-inflammatory response following ablation of TREM2, our data support a contradictory scenario that the sub-acute inflammatory reaction after stroke is attenuated in TREM2-KO mice. We therefore conclude that TREM2 appears to sustain a distinct inflammatory response after stroke.

## Introduction

Cerebral ischemia triggers inflammatory processes which start within hours and last up to weeks [1]–[3]. Resident cells of the innate immune system, predominantly microglia, secrete inflammatory mediators such as cytokines and chemokines, which in turn activate microglial cells and recruit peripheral immune cells [4]. The complex, overlapping and pleiotropic functions of cytokines, chemokines and their receptors may be beneficial as well as deleterious, dependent on the time of release and the affected target cell type [5], [6]. Thus, the inflammatory response after stroke represents an opportunity to influence disease progression and post-stroke recovery.

Post-ischemic phagocytosis of cell debris is crucial for resolving inflammation and reconstitution of tissue homeostasis. Triggering receptor expressed on myeloid cells-2 (TREM2) is a microglial receptor which delivers intracellular signals via the adaptor protein DAP12 [7], [8]. It has been shown to clear apoptotic material by phagocytosis within an anti-inflammatory milieu [9], [10].

Knockdown of TREM2 in microglial cell culture inhibits phagocytosis of apoptotic neurons and increases TNFα transcript expression, whereas overexpression of TREM2 increases phagocytosis and decreases microglial pro-inflammatory responses (TNFα and IL-1β) [11]. Several studies on experimental disease models confirm the anti-inflammatory effect of TREM2-mediated phagocytosis. In a model of experimental autoimmune encephalomyelitis, the transcription of pro-inflammatory cytokines such as TNFα, IFNγ and IL-1β is suppressed by TREM2-transduced myeloid cells, whereas the transcription of anti-inflammatory cytokine IL-10 is increased [12]. Following lipopolysaccharide stimulation, macrophages and bone marrow-derived dendritic cells of TREM2-knockout (TREM2-KO) mice show increased production of pro-inflammatory cytokines such as TNFα, IL-6 and IL-12 p70 [13], [14]. Furthermore, TREM2 knockdown in macrophages increases Toll-like receptor-induced TNF production [15]. In TREM2-deficient animals, colonic mucosal wound repair is delayed and incomplete, associated with an increased expression of pro-inflammatory (TNFα and IFNγ) and a diminished expression of rather anti-inflammatory (IL-4 and IL-13) genes [16]. In a model of Alzheimer’s disease, TREM2 up-regulation is associated with downregulation of TNFα and IL-6 [17]. In contrast, T-cells stimulated with TREM2-expressing microglia cells increase their production of TNFα and CCL2 [18]. In conclusion, the majority of studies demonstrate an anti-inflammatory effect of TREM2 regarding cytokine expression.

The present study focuses on the role of TREM2 in inflammatory processes after stroke. Transient occlusion of the middle cerebral artery (MCAO) – a model that closely resembles human stroke – was used to induce cerebral infarction in mice. Post-ischemic expression of TREM2 and of pro-inflammatory cytokines (TNFα, IL-1α, IL-1β, IL-6), chemokines (CCL3, CCL2, CCL5) and their receptors (CCR1, CCR2, CCR5, CX3CR1) was analyzed via quantitative PCR (qPCR) at different reperfusion times (6 h, 12 h, 24 h, 2 d, 7 d, 28 d). Microglial activation (CD68, Iba1) and CD3-positive T-cell invasion were analyzed via qPCR and immunohistochemistry. The inflammatory mediators were analyzed in TREM2-KO mice and compared with their C57BL/6 littermate controls. Functional consequences of TREM2 knockout on the extent of ischemic injury were assessed by infarct volumetry.

## Methods

### Animals

Two- to four-month-old male C57BL/6 as well as TREM2-KO mice backcrossed at least 10 generations into C57BL/6 (originally obtained from M. Colonna, St. Louis, USA; hereafter termed TREM2-KO) were used [13]. All mice were randomly assigned to the treatment groups and reperfusion times. All animal experiments were approved by the local government (Thueringer Landesamt für Lebensmittelsicherheit und Verbraucherschutz [TLLV], Germany) and conformed to international guidelines.

### Stroke Model

MCAO was performed as previously described [19]. During isoflurane anesthesia (2.5% in a mixture of 3∶1 N_2_O:O_2_), a 7-0 monofilament (70SPRe, Doccol Corp, USA) was introduced into the internal carotid artery through an incision of the right common carotid artery. The middle cerebral artery was occluded for 30 minutes with a range of reperfusion times (6 h, 12 h, 24 h, 2 d, 7 d and 28 d). The effect of surgery was controlled using sham animals at 12 h and at 7 d. Sham animals underwent the same surgical procedure without occlusion of the middle cerebral artery.

### Quantitative PCR (qPCR)

Under deep anesthesia, animals were decapitated and brains were removed. Using a Precision Brain Slicer (BS-2000C Adult Mouse, Braintree Scientific Inc.) coronal sections (2 mm) comprising the infarct (bregma +0.8 and –1.2 mm) were dissected, separated into the ipsi- and contralateral hemisphere and snap frozen. Total RNA was isolated using the RNeasy Lipid Tissue Mini Kit (Qiagen). Equal amounts of total RNA were transcribed into cDNAs (Fermentas). PCR was performed with Brilliant II SYBR® Green QPCR Mastermix (Agilent Technologies) and specific mouse primers at a final concentration of 500 nM (Table S1, [20]). Amplification was performed using Qiagen’s Rotor-Gene 6000 cycler.

TREM2 mRNA expression following stroke was studied in adult C57BL/6 mice that had undergone MCAO with different reperfusion times (6 h, 12 h, 24 h, 2 d, 7 d and 28 d; n = 4 each). TREM2 ratios (ipsi vs contra) were calculated using the Pfaffl equation [21]. The time course of TREM2 expression after stroke was analyzed using one-way repeated measurement ANOVA and post hoc Tukey test for multiple comparisons.

The expression of inflammatory mediators was analyzed in TREM2-KO and littermate control mice at 12 h, 7 d and 28 d after stroke (n = 5/6 each). As important pro-inflammatory cytokines regulated following cerebral ischemia we selected TNFα, IL-1α, IL-1β and IL-6. Microglial activity was analy**z**ed by Iba1 and CD68 transcript expression. To investigate post-ischemic chemokine and chemokine receptor expression CCL3, CCL2, CCL5 and CCR1, CCR2, CCR5, CX3CR1 were analyzed, respectively. External standard curves of purified PCR products were applied for absolute quantification, as described previously [2]. Transcripts were calculated per 1,000 transcripts of Gapdh by including the criterion of the length of each specific amplicon. The statistical analysis was performed by Student’s t test with Benjamini and Hochberg FDR multiple testing correction. Unless otherwise stated, all data are shown as mean ± s.e.m. The levels of significance were set at *p≤0.05; **p≤0.01 and ***p≤0.001.

### Immunohistochemistry

Anesthetized TREM2-KO and littermate control mice were transcardially perfused with 4% paraformaldehyde 7 d or 28 d after MCAO. Brains were removed, post-fixed for 5 hours in 4% paraformaldehyde, cryoprotected in 30% sucrose and finally stored at –80°C. Coronal sections were cut at 40 µm on a freezing microtome (Microm International GmbH, Thermo Scientific). For CD3 staining, TREM2-KO and littermate control mice were used (n = 3, each). Free-floating sections were pre-treated with 0.2% H_2_O_2_ and incubated with rat anti-human CD3 (1∶500, MCA1477T, AbD Serotec) in TBS containing 3% normal donkey serum and 0.2% Triton X-100 at 4°C overnight. Sections were further processed by Vectastain Elite ABC Kit (Vector Laboratories) using a donkey anti-rat biotinylated secondary antibody (712-065-150, Jackson Immunoresearch). Map2 staining was applied to assess the stroke volume in TREM2-KO and littermate control mice (n = 9–14, each). Free-floating sections were pre-treated with 0.2% H_2_O_2_ and blocked in TBS containing 3% normal donkey serum, 2% bovine serum, 3% milk powder, 0.2% Triton X-100 and FAB fragment donkey anti-mouse IgG (1∶100, 715-007-003, Jackson Immunoresearch). Sections were incubated with mouse anti-Map2 (1∶10,000, M1406, Sigma) in TBS containing 3% normal donkey serum and 0.2% Triton X-100 at 4°C overnight. Sections were further processed by Vectastain Elite ABC Kit using a donkey anti-mouse biotinylated secondary antibody (715-065-151, Dianova). For Iba1 immunofluorescence staining, TREM2-KO and littermate control MCAO mice were used (n = 6, each). Free-floating sections were pre-treated with 0.2% H_2_O_2_ and incubated with rabbit anti-Iba1 (1∶100, 019-19741, Wako) in TBS containing 3% normal donkey serum and 0.2% Triton X-100 at 4°C overnight. Immunofluorescent labeling was performed using secondary Cy5-conjugated donkey anti-rabbit (1∶1,000, 711-176-152, Jackson Immunoresearch) for 2 hours. Sections were stained with DAPI (D9542, Sigma) and analyzed with a confocal laser-scanning microscope (LSM 710 Meta, Carl Zeiss AG).

### Quantification of the Infarct Size

The area of the infarct as well as that of each hemisphere (mm^2^) was measured on MAP2 stained brain sections by tracing these regions on the computer screen via a digital camera (Hamamatsu photonics) and Scion Image software (Scion Corporation). The infarct area was quantified on every twelfth section (40 µm). Infarct volumes were calculated as percentage of the ipsilateral hemisphere. Differences of infarct volumes were statistically analyzed by Student’s t test with Benjamini and Hochberg FDR multiple testing corrections (MCAO mice at day 7 and 28: TREM2-KO, littermate controls, n = 9−14, each). The level of significance was set at p≤0.05.

### Quantification of Iba1 and CD3-positive T-cells

Five photographs per animal and condition were taken at defined areas. Images were taken with LSM 710 Meta for Iba1 and with Axioskop 2 (Carl Zeiss AG) for CD3 analyses. Numbers of Iba1 and CD3-positive T-cells were quantified by ZEN (Carl Zeiss AG) and by ImageJ (NIH) software, respectively. Total numbers of Iba1 positive cells were statistically analyzed by Student’s t test with Benjamini and Hochberg FDR multiple testing correction (MCAO mice at day 7 and 28: TREM2-KO, littermate controls, n = 6, each). Total numbers of CD3-positive T-cells in littermate controls 7 d after stroke were taken as 100%. Differences between TREM2-KO mice and their littermates were analyzed by Student’s t test (MCAO mice at day 7 and 28: TREM2-KO, littermate controls, n = 3, each). The level of significance was set at *p≤0.05, **p≤0.01 and ***p≤0.001.

## Results

### Up-regulation of TREM2 in Ischemic Brain Tissue

**Figure 1:**
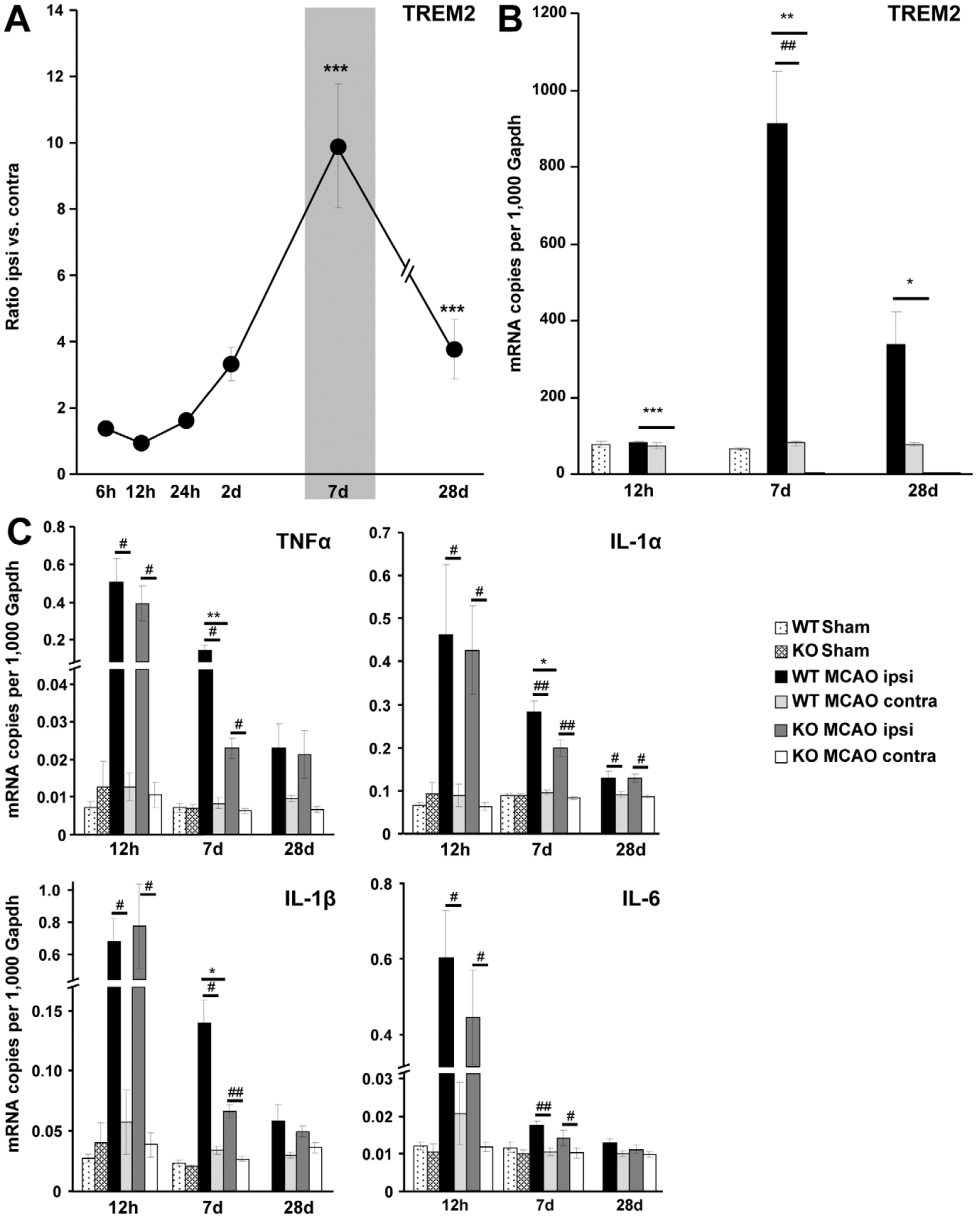
Expression of TREM2 and pro-inflammatory cytokine transcripts in C57BL/6 and TREM2-KO mice after stroke. **A.** Gene transcripts for TREM2 were determined at 6 h, 12 h, 24 h, 2 d, 7 d and 28 d after stroke in the ipsilateral ischemic hemisphere of C57BL/6 mice. TREM2 was up-regulated at 7 d and 28 d after stroke. Data are displayed as mean ± s.e.m. (ratio ipsi vs. contra), n = 4 each, ***p≤0.001. **B.** TREM2 gene transcripts were determined at 12 h, 7 d and 28 d after stroke (MCAO) as well as after sham procedure (Sham) in TREM2-KO mice (KO) and littermate controls (WT). Increased TREM2 gene transcription was detected in the ischemic hemisphere of littermate controls (WT MCAO ipsi) at 7 d and 28 d. No increased gene transcripts for TREM2 were observed after stroke in littermate controls at the contralateral side (WT MCAO contra) and following sham procedure (WT Sham). TREM2-KO mice (KO) did not show any TREM2 gene transcription. **C.** Gene transcripts of cytokines were determined after stroke (MCAO) as well as after sham procedure (Sham) in TREM2-KO mice (KO) and littermate controls (WT). A strong up-regulation of pro-inflammatory cytokines (TNFα, IL-1α, IL-1β and IL-6) was observed 12 h after stroke. A reduced gene transcription of TNFα, IL-1α and IL-1β was observed in TREM2-KO (KO MCAO ipsi) compared to littermate control mice (WT MCAO ipsi) at 7 d after stroke. No differences in cytokine gene transcription were revealed between TREM2-KO mice (KO MCAO ipsi) and littermate controls (WT MCAO ipsi) at 12 h and at 28 d. No increased cytokine expression was observed after stroke in littermate controls at the contralateral side (contra) and in sham operated mice (Sham). Bars represent mean ± s.e.m. (mRNA copies per 1,000 Gapdh), sham: n = 3 each and MCAO: n = 5/6 each, WT ipsi vs. WT contra ^#^p≤0.05, ^##^p≤0.01, WT ipsi vs. KO ipsi *p≤0.05, **p≤0.01, ***p≤0.001.

Stroke was induced in C57BL/6 mice by MCAO and gene transcription of TREM2 was monitored. Cerebral TREM2 mRNA expression was up-regulated in the ischemic hemisphere, showing a peak at 7 d after MCAO (ratio ipsi vs. contra: 10±1.9, p≤0.001, n = 4) and remaining up-regulated at 28 d (ratio ipsi vs. contra: 4±0.9, p≤0.001, n = 4) (Figure 1A). No increased gene transcripts for TREM2 were observed after stroke in littermate controls (WT) at the contralateral side and in sham-operated mice (Figure 1B). TREM2 deficient mice (TREM2-KO) served as controls and showed no gene transcription of TREM2 (Figure 1B).

### Decreased Pro-inflammatory Cytokine Transcription in TREM2-KO Mice after Stroke

Next, we analyzed cytokine gene transcription after stroke by comparing TREM2-KO mice with littermate controls. A strong up-regulation of TNFα, IL-1α, IL-1β and IL-6 was observed at 12 h in the ischemic hemisphere after MCAO. The acute inflammatory cytokine transcription at 12 h reperfusion was very similar between TREM2-KO mice and their littermate controls (Figure 1C). At 7 d after MCAO, transcription of TNFα, IL-1α and IL-1β was reduced in TREM2-KO compared to littermate control mice (Figure 1C). Gene transcripts for TNFα were reduced in TREM2-KO mice to 15% ±1.9, for IL-1α to 69% ±6.9 and for IL-1β to 47% ±4.2 (*p≤0.05, **p≤0.01, n = 5/6 each; Figure 1C). No significant difference in cytokine gene transcription between TREM2-KO mice and littermate controls was observed at 12 h and 28 d after stroke (Figure 1C). Furthermore, no increased cytokine expression was observed after stroke in littermate controls at the contralateral side and in sham-operated mice (Figure 1C). Basal cytokine transcription was found to be unchanged in brain hemispheres of TREM2-KO mice compared to littermate controls (Figure S1). Thus, a reduced gene transcription of pro-inflammatory cytokines was detected in TREM2-KO mice selectively at 7 d after stroke, a time point that also showed the highest TREM2 expression levels in diseased littermate control mice.

### Decreased Microglial Activity in TREM2-KO Mice after Stroke

**Figure 2:**
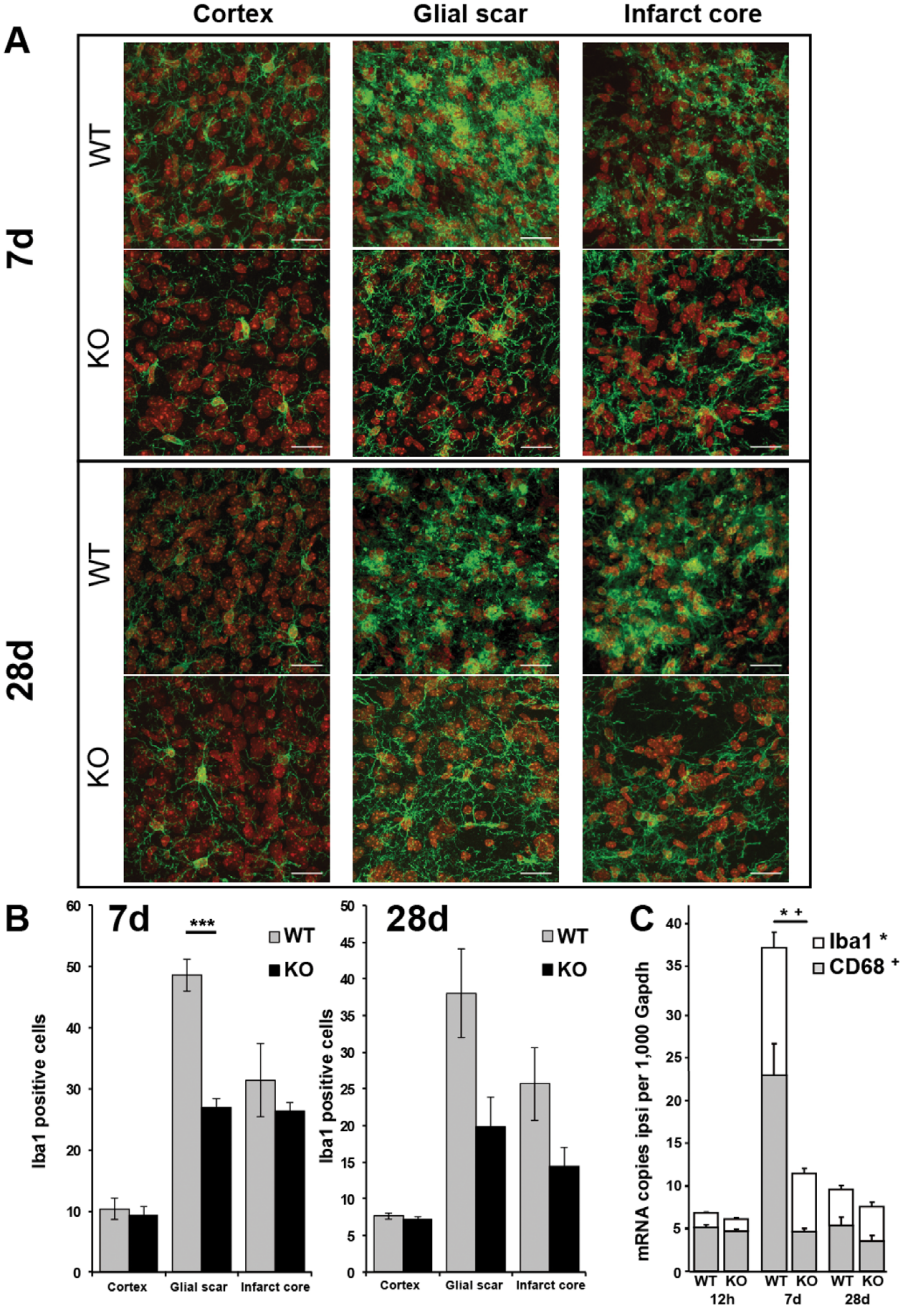
Decreased microglial activation in TREM2-KO mice after stroke. **A.** Post-ischemic Iba1 positive cells (green) displayed the typical activated amoeboid phenotype in the glial scar and infarct core at 7 d and 28 d in WT mice, whereas they remained in a ramified phenotype in TREM2-KO mice. Nuclei of cells counterstained with DAPI (for a better distinction the color was changed to red). Scale bar 20 µm. **B.** Fewer Iba1 positive activated microglial cells were revealed in the glial scar of TREM2-KO mice (KO) at 7 d following stroke compared to littermate controls (WT). Bars represent mean ± s.e.m., n = 5/6 each, WT ipsi vs. KO ipsi ***p≤0.001. **C.** Gene transcripts for Iba1 and CD68 were determined at 12 h, 7 d and 28 d after stroke in TREM2-KO mice (KO) and littermate controls (WT). Attenuated gene transcription of Iba1 and CD68 was observed 7 d after stroke in TREM2-KO (KO) mice compared to control (WT) mice. Bars represent mean ± s.e.m. (mRNA copies per 1,000 Gapdh), n = 5/6 each, WT ipsi vs. KO ipsi *p≤0.05 (Iba1), ^+^p≤0.05 (CD68).

Immunohistochemistry was performed at 7 d and 28 d after stroke to analyze microglial cell activation. Iba1 positive microglial cells in the infarct core as well as in the surrounding glial scar displayed the typical activated amoeboid phenotype in wildtype controls at both investigated times (Figure 2A). However, microglial cells in TREM2-KO mice remained in a more non-activated ramified phenotype with long Iba1 positive processes (Figure 2A). We further quantified the number of Iba1 positive cells in the ipsilateral cortex, the glial scar as well as the infarct core. Significantly fewer Iba1 positive cells were revealed in the glial scar of TREM2-KO mice at day 7 following stroke compared to littermate controls (56% ±6.6, p≤0.001, n = 6 each; Figure 2B). No significant differences in the number of Iba1 positive cells were revealed 28 d after stroke in TREM2-KO mice (Figure 2B).

Next, we performed qPCR analysis for Iba1 and CD68 gene transcripts to confirm the attenuated activation profile of microglial cells in TREM2-KO mice. Both gene transcripts, Iba1 and CD68, were reduced in TREM2-KO mice at day 7 after stroke (KO vs. WT; Iba1∶46% ±3.7, CD68∶20% ±2.0, p≤0.05, n = 5/6 each; Figure 2C), while no significant differences were observed at 12 h and 28 d after stroke (Figure 2C). Basal Iba1 and CD68 gene transcription was unchanged in brain hemispheres of TREM2-KO mice compared to littermate controls (Figure S1).

### Decreased Chemokine and Chemokine Receptor Transcription in TREM2-KO Mice after Stroke

**Figure 3:**
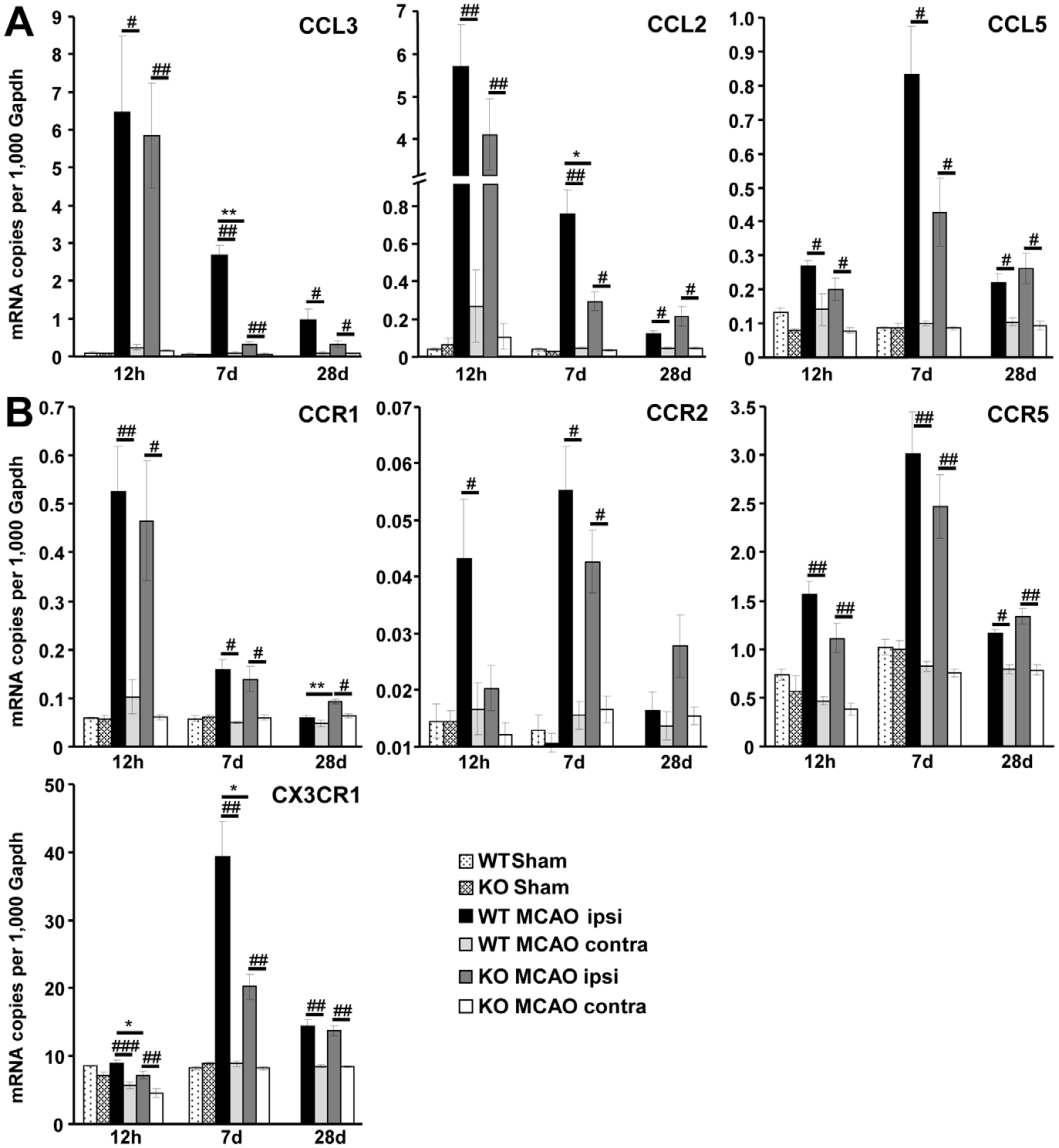
Decreased chemokine and chemokine receptor gene transcription in TREM2-KO mice after stroke. **A.** The mRNAs for the chemokines CCL3, CCL2 and CCL5 were increased following stroke in littermate controls (WT) as well as in TREM2-KO mice, with a peak at 12 h for CCL3 and CCL2 and at 7 d for CCL5. Reduced gene transcription of CCL3 and CCL2 was observed at 7 d after stroke in TREM2-KO mice compared to littermate controls. No increased chemokine expression was observed after stroke in littermate controls at the contralateral side and in sham-operated mice. **B.** Gene transcripts of chemokine receptors were increased after stroke, with a peak at 12 h for CCR1 and at 7 d for CCR2, CCR5 and CX3CR1. Chemokine receptor CX3CR1 expression was attenuated following stroke in TREM2-KO mice. Post-ischemic expression of chemokine receptors was unchanged in littermate controls at the contralateral side and in sham-operated mice. Bars represent mean ± s.e.m. (mRNA copies per 1,000 Gapdh), sham: n = 3 each and MCAO: n = 5/6 each, WT ipsi vs. WT contra ^#^p≤0.05, ^##^p≤0.01, ^###^p≤0.001, WT ipsi vs. KO ipsi *p≤0.05, **p≤0.01.

Since microglial cells showed an attenuated activation profile in TREM2-KO mice, we analyzed typical microglial chemokines known to be involved in the recruitment of leukocytes. Transcripts for the chemokines CCL3, CCL2 and CCL5 were increased in the ischemic hemisphere of wildtype control mice following stroke, with a peak at 12 h for CCL3 and CCL2 and a peak at 7 d for CCL5 (Figure 3A). Whereas all investigated chemokines were found to be unchanged in their acute response at 12 h after stroke (TREM2-KO vs. WT), the increased expression of CCL3 and CCL2 was attenuated in TREM2-KO mice at 7 d after stroke (CCL3: to 12% ±1.3, CCL2: to 37% ±6.5, p≤0.05, n = 5/6 each; Figure 3A).

Transcripts of chemokine receptors were also increased in the ischemic hemisphere of wildtype control mice, with a peak at 12 h for CCR1 and a peak at 7 d for CCR2, CCR5 and CX3CR1 after stroke (Figure 3B). Whereas CCR1, CCR2 and CCR5 expression was found to be unchanged, a reduced gene transcription of the microglial-associated chemokine receptor CX3CR1 was observed after stroke in TREM2-KO mice compared to littermate control mice (12 h: to 79% ±6.7; 7 d: to 50% ±4.6, p≤0.05, n = 5/6 each; Figure 3B). The expression of chemokines and chemokine receptors was unchanged after stroke in littermate controls at the contralateral side and in sham-operated mice (Figure 3). Basal chemokine and chemokine receptor transcription was unaltered in brain hemispheres of TREM2-KO mice compared to littermate controls (Figure S1).

### Fewer CD3-positive T-cells in TREM2-KO Mice after Stroke

**Figure 4:**
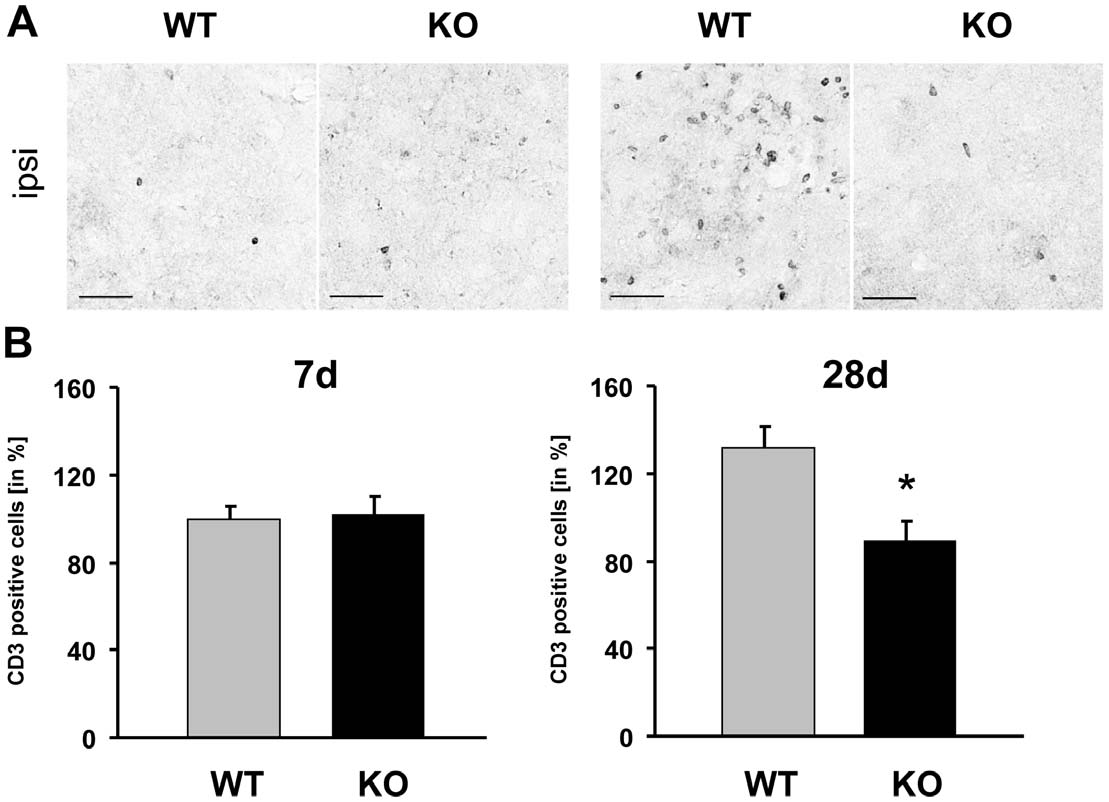
Fewer CD3-positive T-cells in TREM2-KO mice after stroke. **A.** CD3-positive T-cells invaded the infarct core at 7 d and 28 d following stroke. Increased numbers of CD3-positive T-cells were visible in the infarct core 28 d after stroke in littermate controls, but not in TREM2-KO mice. Scale bar 50 µm. **B.** Fewer CD3-positive T-cells were detected 28 d after stroke in TREM2-KO mice compared to their littermate controls. Bars represent mean ± s.e.m, n = 3 each, WT ipsi vs. KO ipsi *p≤0.05.

Next, we investigated whether the attenuated release of chemokines by activated microglial cells in TREM2-KO mice impacts the invasion of leukocytes by determining the number of invasive CD3-positive T-cells immunohistochemically at 7 d and 28 d after stroke. At 28 d following stroke, a strong invasion of CD3-positive T-cells was observed in the infarct core of littermate control mice, whereas such a change was absent in TREM2-KO mice (Figure 4A). At 7 d after stroke, littermate controls and TREM2-KO mice exhibited a similar number of CD3-positive T-cells (100% ±5.72 and 102% ±7.71, respectively). However, 28 d after stroke, the number of CD3-positive T-cells was reduced in TREM2-KO mice (90% ±8.4) compared to littermate controls (132% ±9.3, *p≤0.05, n = 3 each; Figure 4B). Moreover, the amount of CD3-positive T-cells in TREM2-KO mice 28 d after stroke was at a comparable level as observed 7 d after stroke in littermate controls and TREM2-KO mice. Thus, reduced microglial activation and reduced expression of chemokines at day 7 after stroke were followed by a diminished invasion of CD3-positive T-cells in TREM2-KO mice.

### No Change in Brain Tissue Injury in TREM2-KO Mice after Stroke

**Figure 5:**
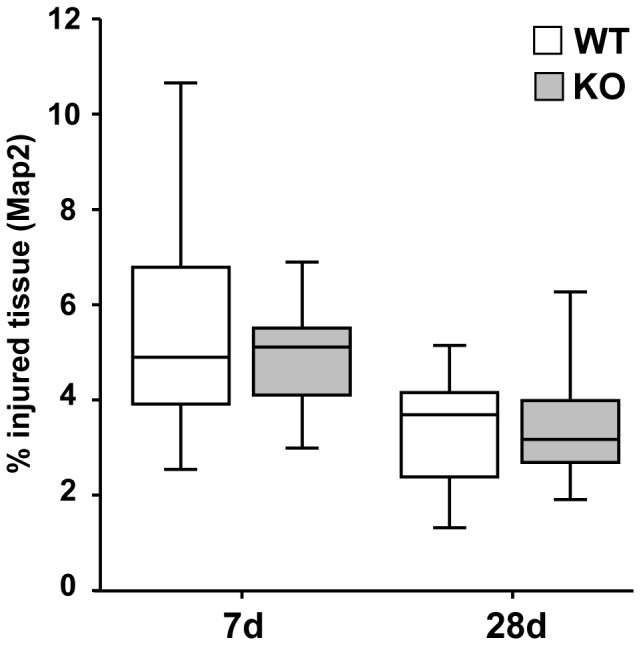
No change in brain tissue injury in TREM2-KO mice after stroke. Infarct volumes of TREM2-KO and littermate control mice were analyzed at 7 d and 28 d based on Map2 immunohistochemistry. TREM2-KO mice and littermate control mice showed the same relative loss in Map2 immunostaining as a sign of tissue injury. Box and whisker plots represent mean ± s.e.m. (mm^3^ injured tissue vs. ipsilateral hemisphere in %), n = 9–14 each.

Immunostaining for Map2 was performed to determine the extent of the lesioned tissue. TREM2-KO mice and their littermate controls displayed comparable lesion sizes at 7 d and 28 d after stroke (mm^3^ injured tissue vs. ipsilateral hemisphere in %; 7 d: WT 4.8% ±0.70, KO 5.1% ±0.35, 28 d: WT 3.6% ±0.39, KO 3.0% ±0.27, n = 9–14 each; Figure 5). Thus, despite an attenuated inflammatory response in TREM2-KO mice, no effect on the lesion size was observed.

## Discussion

TREM2 stimulation induces a signaling cascade resulting in the activation of microglia, which in turn contributes to tissue repair and phagocytosis of apoptotic cellular debris [7], [8], [15]. Thereby, TREM2 sustains immune homeostasis and triggers rather an anti-inflammatory response [9]–[12], [15]. We found an unchanged TREM2 expression at early reperfusion times (hours), but a strong TREM2 up-regulation 7 and 28 days after stroke (up to 10 fold). Thus, TREM2 might play a critical role within the subacute phase after stroke. Induction of the heat shock protein HSP60 stimulates the phagocytic activity of TREM2 expressing microglia [22] and could represent one mechanism of the anti-apoptotic and anti-inflammatory effect of HSP60. Alternatively, post-ischemic HSP60 induction [23] may underlie the dramatic increase of TREM2 following stroke observed here.

To gain more insight into the possible functions of TREM2, we analyzed the post-stroke inflammatory responses in TREM2-KO mice. Inflammatory mediator transcription was unaltered between TREM2-KO mice and littermate controls at 12 h after stroke, indicating a similar acute inflammatory response. This finding complies with our initial results implying that TREM2 plays a somewhat later role after stroke. Indeed, TNFα, IL-1α, IL-1β, CCL2, CCL3 and CX3CR1 transcript expression differed in the ischemic cerebral tissue of TREM2-KO mice compared to littermate controls at day 7 after stroke. Although we initially expected an exaggerated pro-inflammatory response following ablation of TREM2, our data support the contradictory scenario that the inflammatory reaction is attenuated. Though the main cytokines and chemokines revealed an attenuated expression in the sub-acute phase following stroke, chemokine receptors CCR1, CCR2 and CCR5 were found to be unaltered following TREM2 knockout. TREM2-independent expression of CCR5 has previously been reported. Following TREM2 stimulation in dendritic cells, it was found that CCR7 transcription was strongly upregulated whereas CCR5 expression remained unchanged [24]. TREM2 and the chemokine receptors induced by TREM2 stimulation might have different functions in cerebral ischemia. TREM2 and CX3CR1 are both involved in phagocytosis as well as in anti-inflammatory processes. CCR1, CCR2 and CCR5 are mainly migration-inducing receptors that mediate either phagocytotic (CCR1) or anti-inflammatory (CCR2 and CCR5) processes. However, unlike TREM2 and CX3CR1, they are not simultaneously involved in both signaling pathways [25]. Nevertheless, we found that the overall immune response was unexpectedly anti-inflammatory in TREM2-KO mice compared to littermate controls. While TREM2 signaling is anti-inflammatory after confrontation of phagocytes with apoptotic cells [11], TREM2 stimulation by a bacterial trigger induces an inflammatory reaction via release of reactive oxygen species [26]. In comparison to previous animal models and *in vitro* studies on TREM2, one major difference in our study is clear. Cerebral ischemia is associated with massive necrotic cell death that could potentially influence TREM2 signaling via release of reactive oxygen species, as in the case of bacterial stimuli.

Though several studies highlight the deleterious role of the inflammatory response after stroke [27]–[30], it has become widely accepted that cytokines, chemokines and their specific receptors are neither detrimental nor beneficial by themselves [31]–[33]. Cerebral inflammation is complex and most mediators bear overlapping and pleiotropic functions [5], [6]. The effect of inflammatory mediators depends mainly on their expression level, the time and duration of their release, the affected target cells and therefore the pathways that are specifically activated. Activated microglial cells represent one of the most important cellular components of inflammation. Following focal ischemia, microglial cells exert proliferating activity and significantly exceed in number over infiltrating blood-borne macrophages [34]. Moreover, the majority of phagocytes in the infarct area are derived from cerebral microglia, and therefore local defense mechanisms for tissue clearance predominate over immune cells arriving from the blood after ischemic damage [35]. Following stroke, microglial cells might contribute to an exaggerated tissue injury due to inflammation, but might also be important for tissue repair and neuroprotection via phagocytosis [36]. Within this study, we found a decreased transcript expression of CD68 and Iba1 in the ischemic hemisphere as well as fewer Iba1-positive cells in the glial scar of TREM2-KO mice 7 days after MCAO. This attenuated microglial activation was accompanied by a lower occurrence of CD3-positive T-cells in the infarct core of TREM2-KO mice 28 days after stroke. Normally, activated T-cells invading the brain lead to a second wave of inflammation [37]. This would be absent in the TREM2-KO mice which also have a milder inflammation response. Taken together, our data clearly link TREM2 in the sub-acute phase after stroke to an inflammatory response associated with microglial activation, followed by attraction of CD3-positive T-cells at the lesion site. Attenuation of this inflammatory response in the sub-acute phase by ablation of TREM2 had no effect on the lesion size.

### Summary and Conclusion

TREM2 transcript expression was greatly increased in the sub-acute phase after MCAO in mice. At this time, TREM2-KO mice revealed an attenuated cytokine and chemokine expression, accompanied by decreased microglial activation and diminished CD3-positive T-cell infiltration, but an unchanged lesion size. The precise mechanism underlying this attenuated post-stroke inflammatory response in TREM2-KO mice remains to be elucidated. A further question that arises is whether these altered inflammatory processes in TREM2-KO mice have beneficial or deleterious functional consequences following stroke. Future work in our laboratory will therefore involve batteries of functional tests on TREM2-KO mice to analyze their motor and learning behavior following stroke.

## Supporting Information

Figure S1 **No change in the basal gene transcription of inflammatory mediators in the brain of TREM2 deficient mice.** **A.** Cytokine and chemokine gene transcription was unchanged in brain hemispheres of TREM2-KO mice (KO) compared to littermate controls (WT). Data are presented as mean ± s.e.m., n = 5 each. **B.** The transcripts of chemokine receptors and microglial markers (Iba1, CD68) were unaltered in brain hemispheres of TREM2-KO mice (KO) mice compared to littermate controls (WT). Data are presented as mean ± s.e.m., n = 5 each.
(TIF)

Table S1 **Oligonucleotide sequences.**
(DOC)
