# Supplementary figures and images for: Attenuated Inflammatory Response in Triggering Receptor Expressed on Myeloid Cells 2 (TREM2) Knock-Out Mice following Stroke
Source: PLoS One. 2013 Jan 3;8(1):e52982. doi: 10.1371/journal.pone.0052982 (PMC3536811; doi:10.1371/journal.pone.0052982)

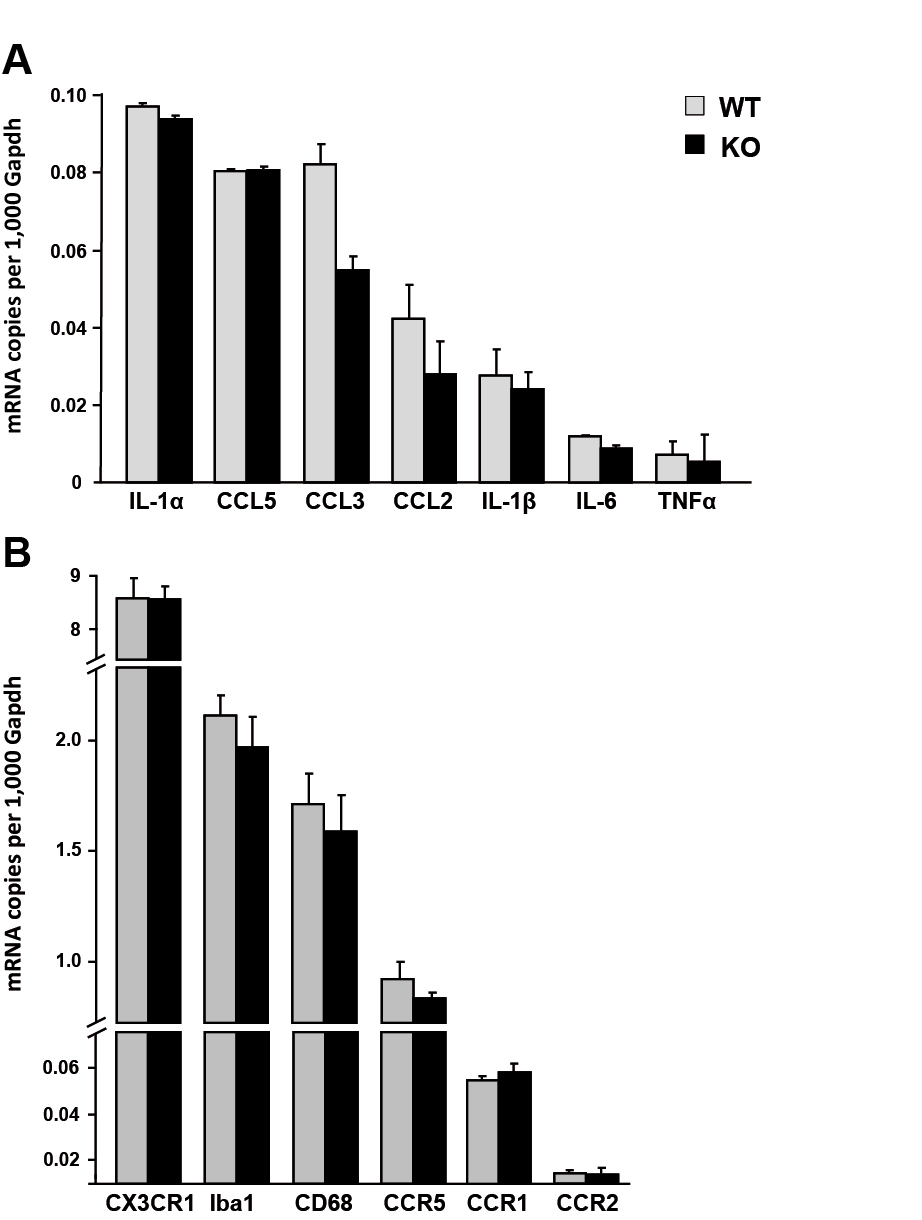

Supplement: Figure S1 — No change in the basal gene transcription of inflammatory mediators in the brain of TREM2 deficient mice. A. Cytokine and chemokine gene transcription was unchanged in brain hemispheres of TREM2-KO mice (KO) compared to littermate controls (WT). Data are presented as mean ± s.e.m., n = 5 each. B. The transcripts of chemokine receptors and microglial markers (Iba1, CD68) were unaltered in brain hemispheres of TREM2-KO mice (KO) mice compared to littermate controls (WT). Data are presented as mean ± s.e.m., n = 5 each. (TIF) [file pone.0052982.s001.tif]
