# Supplementary material for: Attenuated Inflammatory Response in Triggering Receptor Expressed on Myeloid Cells 2 (TREM2) Knock-Out Mice following Stroke
Source: PLoS One. 2013 Jan 3;8(1):e52982. doi: 10.1371/journal.pone.0052982 (PMC3536811; doi:10.1371/journal.pone.0052982)
Supplement: Table S1 — Oligonucleotide sequences. (DOC) [file pone.0052982.s002.doc]

**Table S1.**

Oligonucleotide sequences

| Primer | Sequence (5' - 3') | GenBank accession number |
| --- | --- | --- |
| Gapdh forward | CAACAGCAACTCCCACTCTTC | NM_008084.2 |
| Gapdh reverse | GGTCCAGGGTTTCTTACTCCTT |
| TNFα forward | GTCTACTGAACTTCGGGGTGAT | NM_013693.2 |
| TNFα reverse | ATGATCTGAGTGTGAGGGTCTG |
| IL-1α forward | GCCTTATTTCGGGAGTCTAT | NM_010554.4 |
| IL-1α reverse | TAGGGTTTGCTCTTCTCTTACA |
| IL-1β forward | GAAGAGCCCATCCTCTGTGA | NM_008361.3 |
| IL-1β reverse | TTCATCTCGGAGCCTGTAGTG |
| IL-6 forward | ACAAAGCCAGAGTCCTTCAGAG | NM_031168.1 |
| IL-6 reverse | CATTGGAAATTGGGGTAGGA |
| CCL3 forward | TGGAACTGAATGCCTGAGAGT | NM_011337.2 |
| CCL3 reverse | TAGGAGATGGAGCTATGCAGGT |
| CCL2 forward | AGGTGTCCCAAAGAAGCTGTAG | NM_011333.3 |
| CCL2 reverse | AATGTATGTCTGGACCCATTCC |
| CCL5 forward | CCAGAGAAGAAGTGGGTTCAAG | NM_013653.3 |
| CCL5 reverse | AAGCTGGCTAGGACTAGAGCAA |
| Trem2 forward | GACCTCTCCACCAGTTTCTCC | NM_031254.2 |
| Trem2 reverse | TACATGACACCCTCAAGGACTG |
| Iba1 forward | ACAGCAATGATGAGGATCTGC | NM_019467.2 |
| Iba1 reverse | CTCTAGGTGGGTCTTGGGAAC |
| CD68 forward | TTCTGCTGTGGAAATGCAAG | NM_009853.1 |
| CD68 reverse | GAGAAACATGGCCCGAAGT |
| CCR1 forward | TCCTACTAGGTTGGGACCTTGA | NM_009912.4 |
| CCR1 reverse | GAATCTCCATCCTTTGCTGAG |
| CCR2 forward | AAGGAGCCATACCTGTAAATGC | NM_009915.2 |
| CCR2 reverse | GTTGATAGTATGCCGTGGATGA |
| CCR5 forward | CTAGCCAGAGGAGGTGAGACAT | NM_009917.5 |
| CCR5 reverse | TATAGGTCGGAACTGACCCTTG |
| CX3CR1 forward | TGAGTGACTGGCACTTCCTG | NM_009987.3 |
| CX3CR1 reverse | AATAACAGGCCTCAGCAGAATC |

The GenBank accession numbers were obtained from the NCBI.
